# Women and HIV in a moderate prevalence setting: an integrative review

**DOI:** 10.1186/1471-2458-13-552

**Published:** 2013-06-06

**Authors:** Michelle L Redman-MacLaren, Jane Mills, Rachael Tommbe, David J MacLaren, Richard Speare, William JH McBride

**Affiliations:** 1 School of Medicine and Dentistry, James Cook University, McGregor Rd, Smithfield, Cairns, Australia; 2 School of Nursing, Midwifery and Nutrition, James Cook University, Cairns, Australia; 3 School of Health Sciences, Pacific Adventist University, Port Moresby, National Capital District, Papua New Guinea; 4 Anton Breinl Centre for Public Health and Tropical Medicine, School of Public Health, Tropical Medicine and Rehabilitation Sciences, James Cook University, Townsville, Australia

**Keywords:** Women, Gender, HIV, Papua New Guinea, Integrative review, Moderate prevalence setting

## Abstract

**Background:**

Almost 32,000 people are living with human immunodeficiency virus (HIV) in Papua New Guinea (PNG). The primary route of transmission in this moderate prevalence setting is through heterosexual sex. Thus a gendered understanding of HIV is required to inform HIV prevention, treatment and care options. The aim of this review is to investigate understandings specifically about women and HIV in PNG and to identify gaps in the literature to inform future HIV research.

**Methods:**

An integrative review of literature about women, HIV and PNG was conducted using a systematic search of online databases, including book chapters and grey literature. Prior to inclusion, literature was assessed using inclusion and exclusion criteria, and the Critical Appraisal Skills Programme (CASP) appraisal tool. Selected articles, book chapters and reports were coded and a constant comparative method of analysis used to construct a series of themes.

**Results:**

The 26 articles, book chapters and reports included in the review were predominantly descriptive, original research (23/26 pieces of literature). Six themes were identified in the literature: economic, social and cultural factors (including mobility); gender issues (including violence against women); knowledge about HIV (including perception of risk of HIV); religious beliefs about HIV; women perceived as responsible for HIV transmission; and prevention of HIV. Literature about women and HIV in PNG is predominantly focussed upon women who sell sex, women as mothers or young women. Women are usually represented as either victims of HIV or responsible for transmitting HIV. Anthropological and social research has described the economic, social and cultural context along with the lived experience of HIV in PNG, but there is limited operations research or implementation research available.

**Conclusions:**

The literature reviewed has highlighted the importance of a gendered analysis of HIV prevention, care and treatment in PNG. There is an opportunity for operations, implementation and health systems research about HIV in PNG to shift research from description to action.

## Background

Almost 32,000 people are living with human immunodeficiency virus (HIV) in Papua New Guinea (PNG) in 2012 [1]. HIV in this moderate prevalence setting is predominantly transmitted through heterosexual sex and needs to be understood within a complex and diverse social, cultural and spiritual milieu. A gendered understanding of the HIV epidemic in PNG is required to begin to reduce incidence of HIV transmission in PNG [2-4]. This integrative review examines current literature specifically about women and HIV in PNG, describing themes emerging from the literature and identifying gaps in knowledge which may contribute to successful HIV prevention, care and treatment of women in PNG.

PNG is a Pacific Islands country with a population of almost 7 million and over 800 languages [5,6]. Approximately 48% of the population is under 14 years of age [6]. Most people in PNG live a subsistence lifestyle in village settings, with around 85% located in rural areas [6]. Almost all the population of PNG identify as Christian (96%), with more than 10 mainstream churches, and a number of less well known denominations [7].

PNG has 90% of all reported HIV infections in the Oceania Region [8,9], with an estimated prevalence of 0.79% of the adult population living with HIV [1]. HIV rates across provinces and sexual risk groups are unevenly distributed [9,10]. Current HIV prevalence is estimated from data collected from 203 antenatal clinics across PNG, supplemented with national population and antiretroviral therapy (ART) data [1,11]. At risk populations, including women who sell sex, have higher rates of HIV than the general population, with a recent study of women who sell sex in Port Moresby reporting a HIV rate of 17.8% [12].

Women in PNG occupy a wide variety of roles: from wealthy, urban professional to poor, subsistence farmers. However, the majority of women in PNG live in rural areas and have primary responsibility for food production, with limited access to the cash economy. There are matrilineal societies in PNG (where land is inherited through the mother’s line); however the majority are patrilineal. Educational opportunities for many girls and women are also limited, with approximately 50% of women in PNG considered literate [7]. While there have been some improvements in educational opportunities, only 12.4% of girls complete secondary school, which is half the number of boys who complete secondary school [13]. The life expectancy of a woman in PNG is 65 years compared to men at 62 years [14]. Of the 111 members of the National Parliament in 2012, only three were women [15].

Women in PNG experience maternal mortality rates of 230/100,000, the highest in the Asia Pacific region [16]. However, these rates are challenged by some working in the health sector in PNG, who estimate the rate is closer to 750/100,000 in some areas [17]. Women predominantly engage with the health care system as mothers or expectant mothers, and the challenges faced by HIV positive women are many fold and require a specialist response [18]. HIV transmission from parent-to-child is an avoidable tragedy that contributes to high rates of HIV in PNG. Not all antenatal clinics are equipped to provide antiretroviral therapies (ARTs) to prevent mother to child transmission.

Over two thirds of women in PNG will experience violence at some time in their life, most often perpetrated by their male partner or family member/s [19-21]. PNG is ranked 140th out of 146 countries for gender equality (UNDP 2011), with gender inequality and violence against women increasingly linked with increased HIV risk [22-26].

Sixty percent of people living with HIV in PNG are women (NACS 2012), with younger women over-represented in this group [27]. Significant risk of HIV infection exists for women in PNG because of unprotected heterosexual sex with multiple partners, often from a young age [28,29]. Women experience high levels of untreated sexually transmitted infections (STIs) [10,30], with weak health care delivery systems and surveillance contributing to the limited ability to detect and treat STIs, including HIV [31-33].

HIV prevention has been challenged to date by the limited incorporation of social, cultural and economic drivers of HIV [34]. Anti-condom sentiments are promoted by some Christian groups (although some Christian churches have actively promoted and distributed condoms) [35,36]. The Abstinence, Be Faithful and use a Condom (ABC) HIV prevention messaging is an example of a strategy which does not take full account of women’s social, cultural and economic position. Women are often unable to insist on condom usage in the predominantly patriarchal nature of PNG society, with high rates of violence including sexual violence and polygamous marriages [25].

The aim of this integrative review is to investigate understandings about women and HIV in PNG and to identify gaps in the literature to inform future HIV research in PNG.

## Methods

An integrative review was undertaken to examine public health literature about women and HIV in PNG. The key questions guiding the literature review were:

(i) What peer-reviewed literature exists about women, HIV and Papua New Guinea?

(ii) What is the nature of that literature?

(iii) What is the knowledge base upon which future HIV prevention strategies can be planned?

(iv) What are the research gaps in the peer-reviewed literature?

An integrative review methodology was employed as it allows for the inclusion of experimental and non-experimental research and for data from the theoretical and empirical literature [37]. This design is appropriate for the diverse range of literature published about HIV in PNG. The review consisted of four phases. The first phase was a comprehensive search of *Pub Med* and *Scopus* using four Medical Subject Headings (MeSH): *HIV*; *women*; *female*; *Papua New Guinea* conducted on the 7 March 2012. *Pub Med* comprises 5,632 journals, including all Medline entries for peer-reviewed health and medical journals. *Scopus* is a large database with almost 19,500 peer-reviewed online journals in areas of science, life sciences, physical sciences, medicine and social sciences. These databases were chosen for their comprehensive focus on health, public health and health and social sciences [38,39].

Peer reviewed journal articles constitute one kind of research output and are not always where research activity is reported [40], as is the case in PNG. The second phase was a search of the first 100 websites in *Google* with the same key terms: *HIV*; *women*; *female*; *Papua New Guinea*, undertaken in March and April 2012. The third phase was a search of HIV literature produced by Papua New Guinea Government departments, the PNG National Research Institute and PNG Institute of Medical Research on their respective websites. The fourth phase was a review of literature sourced from colleagues, researchers and academics in PNG and internationally between March and July 2012, with reference lists from these documents checked for literature that might have been missed.

### Inclusion criteria

The following inclusion criteria were applied to the identified articles, book chapters and reports:

(i)   The primary focus of the literature (or a significant section) was about *women* and *HIV* in *Papua New Guinea*

(ii)   Literature was peer reviewed (as described by journals or book publishers)

(iii)   Literature published between January 2004 and July 2012

### Exclusion criteria

(i)   Non-peer reviewed literature was excluded from the review. This included government reports, research institute publications and reviews which were not peer reviewed.

(ii)   Literature focused on funding responses to HIV and HIV control (including Global Fund/Asia Development Bank investments) and Pacific wide surveillance were not included in the review.

(iii)   A National AIDS Council commissioned literature review of HIV research in PNG was identified but not included in this review as it was not peer-reviewed [41].

(iv)   Literature was not included if it was published before 2004. Although literature published before this year is valuable, the nature of the HIV epidemic in PNG changed with the introduction of ARTs in 2004 [42]. This change shifted a HIV diagnosis for many in PNG from an acute, life threatening infection to a chronic disease.

### Classification of literature

The nature of the literature was classified as: a) original research, b) reviews, c) program descriptions and d) commentary/discussion paper using an adapted research identification schema [43]. Original research was further classified as: (i) descriptive; (ii) measurement studies; (iii) operations/intervention research [43]. The Critical Appraisal Skills Programme (CASP) system of appraisal was adopted to appraise the rigour, key methods, credibility and relevance of the diverse literature being considered for inclusion [44].

## Results

**Figure 1:**
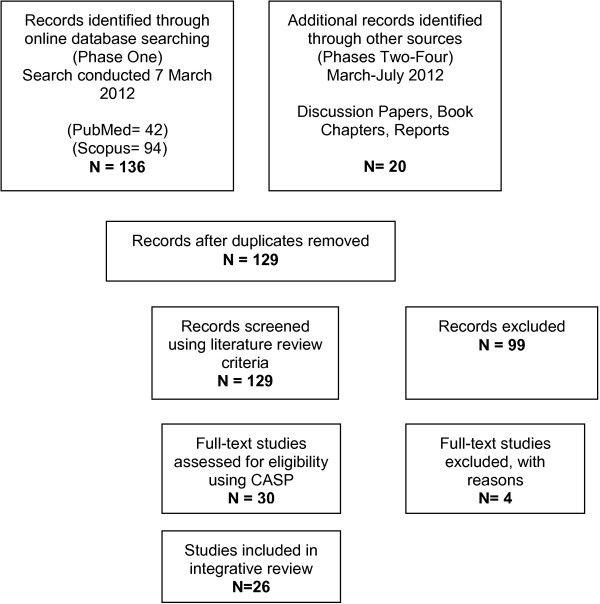
PRISMA flow chart.

Using the identified search terms, *Scopus* returned 94 references and *PubMed* 42 references. Once additional reports, book chapters and reports were identified and duplicates removed, a total of 129 references remained (Figure 1: PRISMA Flow Chart) [45]. The inclusion and exclusion criteria were applied with 26 documents included in the review: 16 journal articles; 4 book chapters; 1 discussion paper; 1 working paper; and 4 reports (bio-behavioural surveys).

The 26 documents were categorised by research type and the full text of these documents analysed by MRM, using the qualitative software programme NVivo™. Codes and themes were then identified using a constant comparative method of analysis. Drawn from grounded theory research methods, constant comparison is a process of comparing codes to codes, codes to themes and themes to themes [46,47]. The qualitative analysis and interpretation was informed by the authors’ reading of the literature, experience in PNG and previous knowledge of issues for women in PNG. NVivo™ was used to organise the outputs of the analysis. All authors further reviewed categorisation of the type of literature and the codes and themes emerging from the literature for this review. The literature included in this review includes: a) original research n = 23; b) program descriptions n = 1; and c) commentary/discussion paper n = 2. There were no reviews identified. Key elements of the 26 documents included in the review are summarised in Table 1 including: author/s, date, source, nature of research, research design and participants; and key findings (Table 1: Summary of Literature).

**Table 1:** Summary of literature

**Title of article/chapter**	**Year of publication**	**Authors**	**Source**	**Nature of research: methods**	**Summary of findings**
1. Why Women Object to Male Circumcision to Prevent HIV in a Moderate-Prevalence Setting	2013	Kelly (Reference No. 70)	Qualitative Health Research	Original Research (descriptive): Qualitative research (semi-structured interviews and focus groups n = 210)	A minority of women accepted male circumcision for the prevention of HIV and other sexually transmitted infections, and for the benefit of penile hygiene and health. Women’s objections to circumcision as a biomedical method of preventing HIV re-emphasise the importance of sociocultural and behavioural interventions in PNG.
2. At risk: The relationship between experiences of child sexual abuse and women’s HIV status in Papua New Guinea.	2012	Lewis (Reference No. 60)	Journal of Child Sexual Abuse	Original Research (descriptive): Mixed methods study (structured survey n = 415; HIV testing n = 312)	Child sexual abuse was reported by 27.5% of the sample (n = 114). Women reporting child sexual abuse were more likely to live in violent relationships, be HIV positive, and have a higher number of sexual partners.
3. HIV knowledge, risk perception, and safer sex practices among female sex workers in Port Moresby, Papua New Guinea.	2011	Bruce (Reference No. 53)	International Journal of Women’s Health	Original Research (descriptive): Mixed Method study (survey n = 174; semi-structured interviews n = 142; focus group discussions n = 32)	Most female sex workers were aware of the risks of HIV but used condoms inconsistently. Contextual barriers to safer sex practices exist. Application for HIV prevention strategies.
4. Understanding the context of providing HIV prevention and treatment in Papua New Guinea.	2011	Clarke (Reference No. 61)	Journal of Trans cultural Nursing	Discussion Paper	Examination of biological, socio cultural, and political influences on the HIV epidemic and on prevention and treatment strategies in PNG.
5. Askim na save (Ask and understand): People who sell and/or exchange sex in Port Moresby.	2011	Kelly (Reference No. 12)	Papua New Guinea Institute of Medical Research and University of New South Wales	Original Research (descriptive): bio-behavioural study using mixed methods (survey n = 593 (women n = 441); in-depth interviews n = 25 (women n = 16)	The study maps the sale and exchange of sex in Port Moresby providing a more detailed understanding of sex workers and their vulnerability to HIV.
6. Reading generalised HIV epidemics as a woman	2011	Reid (Reference No. 2)	State Society and Governance in Melanesia, Australian National University	Discussion paper 2011/4	Socially constructed spaces of femininity and masculinity, including use of power and gendered practices shape interactions. Reading generalised epidemics as a woman provides ways to work within these spaces so that women’s lives and the lives of those that are important to them are transformed and protected.
7. Bio-behavioural sentinel surveillance survey among women attending the Port Moresby General Hospital Antenatal (PPTCT) Clinic 2008	2010 (a)	Arawafu (Reference No. 58)	National Research Institute of PNG	Original Research (descriptive): bio-behavioural study using mixed methods (survey n = 300; women n = 172).	Documented economic, social and cultural factors including sexual practices which contribute to HIV risk for women attending the Port Moresby General Hospital Antenatal Clinic.
8. Bio-behavioural sentinel surveillance survey among women attending Lae Friends STI clinic 2008.	2010 (b)	Arawafu (Reference No. 67)	National Research Institute of PNG	Original Research (descriptive): bio-behavioural study using mixed methods (survey n = 307 women).	Documented economic, social and cultural factors including sexual practices which contribute to HIV risk for women attending the Lae Friends STI Clinic.
9. Behavioural surveillance research in rural development enclaves in Papua New Guinea: A study with the Oil Search Limited workforce.	2010	Buchanan (Reference No. 66)	National Research Institute of PNG	Original Research (descriptive): bio-behavioural study using mixed methods (survey n = 299; women n = 161).	Documented economic, social and cultural factors including sexual practices which contribute to HIV risk in the WR Carpenter Estates workforce, Western Highlands Province.
10. Knowledge, attitudes, practices and behaviour of female sex workers in Port Moresby, Papua New Guinea.	2010	Bruce (Reference No. 50)	Sexual Health Journal	Original Research (descriptive): Quantitative study (n = 79)	72% respondents consistently use condoms. Condom use is yet to reach the required level to protect female sex workers from HIV.
11. AIDS and ‘building a wall’ around Christian country in rural Papua New Guinea.	2010	Dundon (Reference No. 62)	Australian Journal of Anthropology	Original Research (descriptive): Ethnographic enquiry (n = NA)	A growing divide between rural and urban Gogodala, then, has become a major part of the local dialogue about AIDS and represents significant contestation over the practices and ideational basis of Christian country.
12. From Gift to Commodity . . . and Back Again: Form and Fluidity of Sexual Networking in Papua New Guinea	2010	Hammar (Reference No. 54)	In, Civic Insecurity: Law, Order and HIV in Papua New Guinea.	Original Research (descriptive): Ethnographic enquiry (n = NA)	Sexual networking is a social process, thus critical ethnographic and social research provides evidence to respond to HIV in PNG; epidemic in PNG is unique to PNG context; just governance and a vibrant civil society is required to respond successfully to HIV; state sponsored brothels will not be the answer to PNG’s HIV epidemic and fails to respond to male sexual privilege and sex-negative attitudes.
13. Witchcraft, Torture and HIV.	2010	Haley (Reference No. 69)	In, Civic Insecurity: Law, Order and HIV in Papua New Guinea.	Original Research (descriptive): Ethnographic enquiry (n = NA)	Accusations of witchcraft in the Highlands of PNG appear to be increasing and often result in torture, rape and sometimes death. Changed sexual practices shape the way people are experiencing the HIV epidemic in the Highlands, with AIDS-related deaths being widely interpreted in terms of witchcraft.
14. Gendered talk about sex, sexual relationships and HIV among young people in Papua New Guinea.	2010	Kelly (Reference No. 56)	Culture, Health and Sexuality journal	Original Research (descriptive): Qualitative study with high school students (focus groups n = 8; no. student’s =73)	When discussing sex, young men used explicit language and referred specifically to sexual organs and activities; young women did not. Young men were more open publically about sex; young women discussed sex one-on-one and in private. Application for HIV prevention strategies.
15. Attitudes to HIV testing among carers of children admitted to Port Moresby General Hospital, Papua New Guinea.	2008	Allison (Reference No. 57)	Journal of Paediatrics and Child Health	Original Research (descriptive): Qualitative study: semi-structured interviews (n = 40)	Three quarters of the women interviewed would consent to having their child tested for HIV; over half of the women who had never undertaken a HIV test would agree to be tested.
16. Buying Betel and Selling Sex: Contested Boundaries, Risk Milieus, and Discourses about HIV/AIDS in the Markham Valley, Papua New Guinea	2008	Beer (Reference No. 68)	In, Making Sense of AIDS: Culture, Sexuality, and Power in Melanesia	Original Research (descriptive): Ethnographic enquiry (n = NA)	Analysis of demographic and social change for people living in the Markham Valley as conditions for risk of HIV. Specifically, land pressure, population growth and inward migration of non-Wampar people are described.
17. Fear and Loathing in Papua New Guinea: Sexual health in a nation under siege.	2008	Hammar (Reference No. 64)	In, Making Sense of AIDS: Culture, Sexuality, and Power in Melanesia	Original Research (descriptive): Ethnographic enquiry (n = NA)	Strengths and weaknesses of health services in PNG are identified, including limitations of current HIV prevention initiatives. Description of attitudes towards sexual practices and taboos, including treatment of people living (and dying) with AIDS.
18. Mobility, violence and the gendering of HIV in Papua New Guinea.	2008	Lepani, K. (Reference No. 34)	Australian Journal of Anthropology	Original Research (descriptive): Ethnographic enquiry (n = NA)	The links between gender, sexuality and violence hold serious implications for HIV transmission and its social and economic effects.
19. Violence against women in Papua New Guinea.	2008	Lewis (Reference No. 22)	Journal of Family Studies	Original Research (descriptive): Mixed methods study (structured survey n = 415; HIV testing n = 312)	Programmes concerned with HIV prevention must include interventions to counter domestic violence and increasing the social status of women in PNG.
20. Warrior women, the holy spirit and HIV/AIDS in Rural Papua New Guinea.	2007	Dundon (Reference No. 63)	Oceania Journal	Original Research (descriptive): Ethnographic enquiry (n = NA)	Warrior women seek to make both AIDS and those who encourage or enable its spread more visible. A small number of them are overcome by the Holy Spirit - their behaviour increasingly characterised by childishness and uncontrolled sexuality.
21. Knowledge, morality and ‘Kastom’: SikAIDS among young Yupno people, Finisterre Range, Papua New Guinea.	2007	Keck (Reference No. 13)	Oceania Journal	Original Research: Qualitative study: semi-structured interviews (n = 24)	Local understandings of HIV are shaped by cultural, moral and religious concepts based on social values and practices. A broad and contextually sensitive approach to sexual health is required.
22. Men’s extramarital sexuality in rural Papua New Guinea.	2007	Wardlow (Reference No. 55)	American Journal of Public Health	Original Research (descriptive): Qualitative study: interviews (n = 65)	Married women in rural PNG are at risk of HIV primarily because of their husbands’ extramarital relationships. Labour migration puts these men in social contexts that encourage infidelity. Interventions that promote fidelity will fail in the absence of a social and economic infrastructure that supports fidelity.
23. ‘Mainstreaming’ HIV in Papua New Guinea: Putting gender equity first.	2006	Seeley & Butcher (Reference No. 65)	Gender and Development Journal	Programme Description	A scheme in the oil palm industry in PNG that specifically targets women to ensure that they benefit in the harvesting of oil palm. Women are gaining economic independence. The scheme is also reducing conflict and gender-based violence contributing to arresting the spread of HIV.
24. High Prevalence of Sexually Transmitted Infections Among Female Sex Workers in the Eastern Highlands Province of Papua New Guinea: Correlates and Recommendations.	2005	Gare, J (Reference No. 30)	Sexually Transmitted Diseases Journal	Original Research (descriptive): Quantitative study: structured interviews (n = 211)	None of the women were positive for HIV. 74% were positive for at least 1 STI and 43% had multiple STI infections. High-risk sexual behaviours were common among the women, including low and inconsistent use of condoms, with most of them attributing this to unavailability, dislike by or familiarity with clients, and being drunk and/or high on marijuana.
25. “Everything has come up to the open space”: Talking about sex in an epidemic	2005	Lepani (Reference No. 76)	Working Paper No. 15; Australian National University	Original Research (descriptive): Ethnographic enquiry (n = NA)	The Trobriand islands’ context described shows effective communication about HIV needs to centralise local understandings of gender, sexuality and reproduction.
26. Anger, Economy, and Female Agency: Problematizing “Prostitution” and “Sex Work” among the Huli of Papua New Guinea	2004	Wardlow (Reference No. 51)	Signs Journal	Original Research (descriptive): Ethnographic enquiry (n = 18)	Huli women known as *pasinja meri* (passenger women) sell sex not due to material necessity but from anger and resistance. *Pasinja meri*’*s* exchange of sex for money is not perceived as the crude sale of something that should not be sold, but a kind of theft (and consumption) of a resource that rightfully belongs to a woman’s kin.

Before continuing to an analysis of this literature, we wish to acknowledge the conceptual challenge of isolating *women* as a category for this review. Feminist theory has contributed an understanding of sex and gender which separates physical characteristics (sex) from socially ascribed roles (gender) [48]. From a public health perspective the focus of literature reviewed for this article is *women* as a gendered category, understood in the diverse sociocultural context of PNG. Wingood and DiClemente [49] explain that it is gender-based inequality and disparities in expectations which generate risk of HIV for women. This risk for women will be explored within the geographic bounds of PNG.

Throughout the literature included in this review, women were mostly studied in groups depending upon a role they held in society: women who sell or exchange goods for sex [12,30,50-55], young women [13,56] and women as mothers/carers [57,58]. These roles were often the criteria for inclusion in a study. Specific experiences were also examined, such as violence, accusations of witchcraft and sorcery [59] and sexual abuse [22,34,60]. Major themes identified from inductively coding the literature were:

•Economic, social and cultural factors impacting HIV, including mobility

•Gender-based inequality, including violence against women

•Knowledge about HIV, including perception of risk of HIV

•Religious beliefs about HIV

•Women perceived as responsible for HIV transmission

•Prevention of HIV

### Economic, social and cultural factors impacting HIV, including mobility and HIV risk

The changing economic, social and cultural context of PNG is reported to influence the risk of HIV for women. Urbanisation and modernisation result in changed economic, social and cultural conditions, including changed sexual practices. Separation from traditional lands and forms of subsistence (such as gardens) means services in exchange for goods or money is required for survival. For women in urban and peri-urban environments in PNG where there are very limited employment opportunities, the selling or exchanging of sex for money or goods is often ‘survival sex’ [51]. Bruce et al. [53] argue that economic deprivation is likely to influence sexual behaviours which increase the risk of HIV infection. Clark and colleagues support this position, stating that the selling of sex results from a high and increasing population density along with a rapid increase in poverty [61]. Changing economic drivers in PNG are leading to increased mobility for men and women, bringing new social and cultural norms and mores and an associated increased risk of HIV transmission. Lepani states that in contemporary PNG, “where men and women move, money moves as well” [34]. The Highlands Highway was a particular focus of a study by Gare et al. [30] who found that while the highway serves as the major economic route, it may also be the main conduit for the transmission of STIs and HIV between provinces. Wardlow in her work on men’s extramarital sexuality in rural PNG, also highlighted the link between economic drivers and HIV saying: “HIV/AIDS prevention policies and programs should specify and target the socioeconomic structures that make the choice of extramarital sex so likely” [55]. Men and women who live in urban areas and then return to rural villages are also seen as responsible for bringing HIV, creating a social schism between those who travel away to work and those who stay in the village [62]. This dynamic was similarly described in the Yupno region of PNG, with Keck quoting a young man as saying, “the men come and go…and bring this disease (HIV) here into the village” [13].

Cultural factors such as traditional beliefs around sexuality and reproduction, bride price and the inability to discuss sexuality and HIV prevention openly are contributing to increased risk of HIV for women in PNG. Cultural understandings of sexuality and reproduction are discussed at length in a number of the articles reviewed [13,34,55,56,62-64]. Cultural practices such as bride price can increase women’s risk of HIV by placing men in a position of power over sexual decision making. Bride price is an exchange of goods (increasingly, including cash) that is paid to the wife’s family by the husband’s family. Some men whose family pay a bride price expect their wives to practice fidelity but do not expect their wife to be their only sexual partner since they were the givers of bride price. Reporting research from the Highlands of PNG, Wardlow describes *pasinda meri* (literally passenger woman in *Tok Pisin*) as a woman who doesn’t belong to anyone or who has given up her social and cultural roles [55]. Usually this results in selling sex or exchanging goods for sex for survival [51]. Becoming a *pasinda meri* is often a consequence of a woman’s anger at their treatment within cultural systems of bride price and compensation. Being a *pasinda meri* results in a freedom to live outside of cultural norms and while an expression of a woman’s agency it often results in stigmatisation, repudiation and physical attacks by both men and women [51,55,64]. Kelly et al. report the gendered nature of talk about sex, with young men using explicit language about sex in public spaces, while young women discuss sex one-on-one in private indicating the different ways young women and men discuss sex in PNG [56].

### Gender based inequality, including violence against women

Women in PNG experience gender-based inequality, including less access to economic, education, legal and employment opportunities than men [61]. It was reported that women were often unable to negotiate safe sexual practices with their regular partners (including condom use and fidelity). It is also difficult for women to leave high risk relationships [61]. This inequality impacts women’s exposure to HIV and thus the epidemiology of HIV in PNG [34,65]. Physical violence was experienced by 58% of the female sample in one study [22]. Violence not only exposes people to HIV transmission, but can also be a consequence of living with the virus. Rape is a likely contributing factor to the spread of HIV in PNG, with instances of *lainups* (gang rape) common [34]. A study led by Gare reported that 21% of the 210 women interviewed had been the victim of a *lainup*[30]. In PNG, there is a statistically significant link between violence against women and HIV transmission, including sexual abuse in intimate partner relationships, making the improved status of women in PNG a priority area for HIV prevention action [22,65].

### Knowledge about HIV, including perception of risk of HIV

There is a range of findings about women’s knowledge about HIV represented in the literature, with women who sell sex, young women and mothers the focus of studies that include this topic. The majority of women who sell sex or exchange sex for goods know HIV transmission occurs through sexual intercourse [50,58,66,67], although some young people lack adequate knowledge of HIV, including not knowing HIV is an STI that can occur within marriage or a stable relationship [13,56]. HIV is associated with sex and referred to as *sik blong koap* (which translates as ‘sexually transmitted infection’) [56]. On the whole, HIV is not believed to be associated with sexual relationships with regular partners or within marriage, with many believing condoms are not needed in relationships where trust is established [34,53,56,62]. Often, HIV is understood to come from outside the area in which one lives, by people who don’t belong to the area or from those who belong to the area but who have travelled outside and brought HIV back [13,62,68]. In PNG, sickness is commonly understood as a result of wrongdoing, embedding the virus within complex social, cultural and spiritual understandings [13,34,63,69]. Links are made in the Southern Highlands between apparent witchcraft and HIV. Hayley reports AIDS produces the kind of dying that “lend[s itself] to witchcraft accusations”, which can result in torture of accused women and, on occasions, early death [69]. Knowledge of HIV may influence choices about HIV testing [57], but rarely changes sexual behaviour, as many women in PNG are in unequal power relations and have limited power to negotiate safe sex. This lack of agency is common amongst married women [54]. As Kelly and her colleagues remind us, a woman’s risk is far more dependent upon her ability to make and act upon decisions than knowing about HIV and its transmission [56].

### Religious beliefs about HIV

The dominance of Christianity influences the way women experience HIV in PNG. Combined with the effects of colonisation, Christianity preserves beliefs about unequal gender roles and expected sexual behaviours of women [34]. One informant told Dundon, “In the beginning God created man and woman so that plan is in order, it’s in place” [62]. Christian churches have greatly influenced understandings of HIV and AIDS, as *SikAIDS*, as it is known in *Tok Pisin*, is linked with immoral behaviour, promiscuous sex and prostitution [13]. *SikAIDS* is described variously by Christians as a punishment from God, divine retribution, or a call to relinquish sinful sexual practices [13]. Most importantly, condom use is not accepted by some Christian churches as condoms are seen as a mechanism to elude God’s punishment for immoral sexual practices [55]. This rationale extends to male circumcision for HIV prevention. Some women explained that male circumcision is incongruent with Christian principles because it enables promiscuous behaviour and spoils the body (the temple) created by God [70].

### Women perceived as responsible for HIV transmission

A dichotomy is present in the literature about women living with HIV in PNG, with women divided into passive victims (the ‘mothers’) and promiscuous vectors (women who sell sex) [34]. Lepani theorises that women are either represented as victims of HIV or responsible for the spread of HIV. To reduce their risk of being a victim of sexual violence and thus of being infected with HIV, women are expected to stay inside, safe, sedentary and secluded with the inference being that if this code of behaviour is not observed, “rape is the consequence of women being out of place” [34]. Regrettably, research findings fail to support the idea that staying at home ensures safety from sexual assault with Lewis et al. reporting that 44.5% women in a study had experienced sexual abuse perpetrated by their intimate partner [22].

Women seen as responsible for the spread of HIV in the literature are mostly women who sell sex based in the Highlands and PNG’s capital, Port Moresby [30,50,53,61]. Women who sell sex are known as *pamuk meri* or *pasin pamuk* (prostitute) or *tukina meri* (literally a woman who sells sex for 2 Kina, the currency of PNG) [54]. Women who sell sex are often not able to insist upon condom use (including with their intimate partners) and are frequently punished for selling sex by rape, including by gang rape [30,53]. There is also evidence that women who leave their husbands due to rape and violence are also deemed HIV vectors and thus responsible for spreading HIV [54,55]. In Western Province, senior women considered to possess special spiritual powers can identify and enact retribution upon women who are deemed a HIV vector (including divorced women and women who sell sex). This retribution can take the form of social, cultural or spiritual isolation and, on occasions, physical punishment. [30,50,53,62]. Women living with HIV are also often accused of being a *pasin pamuk* or *pamuk meri*[55]. This attitude is often reflected in the way health services are provided (or not) to women, with expressions of stigma reported [64].

### Prevention of HIV

A variety of ideas about HIV prevention are explored in the literature reviewed. These include: encouraging young people to adhere to *kastom* and church requirements [13,34]; patrolling the community and figuratively “building a wall” around an area [62,63]; and, increasing HIV education opportunities and access to condoms [30,50,53]. Seeley and Butcher discuss an example of increased economic and social agency of women and how this may reduce risk of HIV acquisition, because of women’s enhanced bargaining position within the household. These findings demonstrate the importance of expanding a structural and gendered understanding of HIV in PNG as a method of developing strategies for HIV prevention [65].

## Discussion

Women were typically represented in the literature in this review as victims or vectors, with many of the articles in the review portraying women as victims of their social, cultural and religious context. Social and cultural changes such as urbanisation and increased mobility have increased HIV risk for women [55,62]. A more inclusive, contextually sensitive research agenda, addressing gender-related vulnerability is required. A key recommendation from the *United Nations General Assembly Special Session* (*UNGASS*) *2010 Report* was to scale up research on the most-at-risk populations in PNG, which includes women selling sex [1]. There is clearly evidence that this research needs to be conducted. However, there needs to be a reduced focus on constructed categories which serve to reinforce the perceptions of women as victims or vectors and a greater consideration of structural factors (including economic factors) which contribute to gender-related vulnerability of HIV [27,34].

### The nature of the literature

The majority of original research in this review is descriptive research about women and HIV in PNG, with no operations or intervention research. Operations research (also known as operational research) is defined as research which aims to “develop solutions to current operational problems of specific health programmes or specific service delivery components of the health systems” [71], while implementation research aims to “develop strategies for available or new health interventions in order to improve access to, and the use of, these interventions by the populations in need” [71]. There is debate in the literature about the similarities and differences between operations research and evaluation. The intent of both is to systematically collect and analyse program or project data. However, operations research differs from evaluation in that the purpose of operations research is to influence practice and policy [72], whereas the primary focus of an evaluation is to provide information which will (i) improve a product or process (formative evaluation) or (ii) report upon the effectiveness or impact of the project or program (summative evaluation) to generate knowledge about good practices [73].

Limited literature about the impact or outcomes of strategies implemented by government departments or non-government organisations addressing HIV at a programmatic level is published in the peer-reviewed literature. Health services can be unhelpful or dysfunctional [64]. There are few positive accounts of successful HIV prevention activity in the peer-reviewed literature, with Seeley and Butcher’s article being an exception [65]. The exclusion of non-peer reviewed government and non-government organisations reports accounts for some of the gaps identified in this review. While descriptive research is required to develop a deep understanding and an evidence base for action, research about woman and HIV in PNG needs to progress to action-oriented operations and intervention research in order to provide policy makers with evidence for decision making. The Global Fund to Fight AIDS, Tuberculosis and Malaria recommend a minimum of 5-10% of HIV program funds be used on monitoring and evaluation activities, including operations research [74], indicating there is an opportunity for operations, implementation and/or health system research with and for women in PNG about HIV to shift from description to action.

### The research gaps

In addition to the lack of operations or intervention research, there are still a number of gaps in the descriptive research available. Current prevalence estimates are mostly based on HIV identified in antenatal clinics in PNG. We need to know more about PNG women outside the context of antenatal clinics or selling sex [1]. Unequal power between men and women reduces the bargaining abilities of women, including in long-term sexual relationships [53]. In PNG, some women describe feeling offended when men ask to use condoms when having sex with them in the context of marriage or long-term relationships [75]. Additionally, women requesting a condom to be worn in the context of such relationships fear they will be perceived as a *tukina meri*[75]. More research is required to understand these relational dynamics and their impact upon HIV risk in PNG. There is limited research about women and HIV risk in the matrilineal societies in PNG, with the exception of Lepani’s work in the Trobriand Islands [34,76]. Do women in matrilineal societies experience the drivers of HIV differently to those in the Highlands for example? Does land ownership by women contribute to a reduced HIV risk? How is this being impacted by modernity and development? The economic inequity that is described in the literature would be better understood with operations or intervention research being conducted to address social, cultural and/or economic issues to enhance HIV prevention in PNG.

In the light of this review about women, it is clear the role of masculinity as perceived and enacted in PNG is important for HIV prevention in PNG. A renewed focus is required “on masculinities and policy and program frameworks that integrate the issues of male sexual privilege and gender violence” [34]. Male privilege, expressed by violent manifestations of male power needs to be challenged. Richard Eves’ research about masculinity in PNG, which is manifested by men perpetrating violence, is an example of the growing understanding of the social, cultural and spiritual drivers of violence [3]. Research about masculinity in PNG needs to be expanded while remaining contextualised. Some answers may lie in work being done in Vanuatu by Sister Lennon and colleagues, who assist men to consider women as mothers and sisters to connect them with the ‘feelings’ of a rape victim [2].

Limited literature in this review focused on the experience of women living with HIV and AIDS or the experience of women caring for a HIV positive person/people. Stigma against people living with HIV was reported [67]. Women bear the burden of caring for people living with HIV around the globe, with PNG no exception. There are program activities being conducted and reported upon in PNG in forums such as the PNG Medical Symposium (2012; 2013). However, there is opportunity for further operations research to be conducted with people living with HIV in PNG [27].

### Knowledge that can be built upon for future HIV prevention

Literature included in this review provides knowledge about the context of women’s lives in PNG, the gender issues they face, their understanding of HIV and the way women are perceived in relation to HIV. The range of findings about women’s knowledge of HIV reflects the way in which these research findings were generated: from internationally sanctioned knowledge, attitude and practices questions to sociocultural focused, geographically bound ethnographic methods. The current focus on strengthening the capacity of researchers from PNG to undertake HIV research will further enhance the nature and amount of HIV research in PNG [27].

Peer-reviewed literature provides evidence of explicit research methods, results and recommendations which are less able to be influenced by organisational and government agendas [77]. However, the peer review process and the claim of quality has its’ critics, particularly with the advent of a plethora of open access journals and availability of reports and other information online [78]. Peer-reviewed literature about women and HIV in PNG is a useful source of evidence. However, much valuable, nuanced and important evidence for decision making is available in the grey literature. This is demonstrated by the non-peer reviewed systematic literature review of HIV literature in PNG by King and Lupia, which contributed an important synthesis of literature about HIV in PNG [41]. Key recommendations from this review have been instructive for the HIV response in PNG. Recommendations included: increasing the involvement of churches in the HIV response, being guided by culture, working with groups living with or at higher risk of HIV and providing women with more options. In addition to information and behaviour change, more focussed support was recommended for the public sector and researchers [41].

#### Limitations to the review

A limitation of this review is the exclusion of non-peer reviewed literature. There is much valuable information about HIV and prevention options. As identified above, the veracity of this literature is difficult to determine and thus has not been included.

## Conclusions

Women in PNG have the right to feel safe, have choices about HIV prevention, care and treatment and reach their potential in the context of shifting social, cultural and economic conditions of this moderate prevalence setting. The literature reviewed has highlighted the importance of a gendered analysis of HIV prevention, care and treatment in PNG. Opportunities exist for researchers, in partnership with communities, service providers and policy makers to move from descriptive to action-oriented research in order to reduce HIV in PNG.

## Competing interests

The authors declare there they have no competing interests.

## Authors’ contributions

MRM: Conceived of and designed the literature review, acquired and analysed the literature, drafted and edited the manuscript. JM: Provided support for the literature review process, revised the manuscript for important intellectual content and reviewed the final manuscript. RT: Provided literature and advice on sources, revised the manuscript for important intellectual content and reviewed the final manuscript. DM: Provided literature and advice on sources, revised the manuscript for important intellectual content and reviewed the final manuscript. RS: Provided support for the literature review process, revised the manuscript for important intellectual content and reviewed the final manuscript. WJHM: Provided support for the literature review process, revised the manuscript for important intellectual content and reviewed the final manuscript. All authors read and approved the final manuscript.

## Pre-publication history

The pre-publication history for this paper can be accessed here:

http://www.biomedcentral.com/1471-2458/13/552/prepub
